# Tissue-engineered smooth muscle cell and endothelial progenitor cell bi-level cell sheets prevent progression of cardiac dysfunction, microvascular dysfunction, and interstitial fibrosis in a rodent model of type 1 diabetes-induced cardiomyopathy

**DOI:** 10.1186/s12933-017-0625-4

**Published:** 2017-11-02

**Authors:** Masashi Kawamura, Michael J. Paulsen, Andrew B. Goldstone, Yasuhiro Shudo, Hanjay Wang, Amanda N. Steele, Lyndsay M. Stapleton, Bryan B. Edwards, Anahita Eskandari, Vi N. Truong, Kevin J. Jaatinen, Arnar B. Ingason, Shigeru Miyagawa, Yoshiki Sawa, Y. Joseph Woo

**Affiliations:** 1 Department of Cardiothoracic Surgery, Stanford University School of Medicine, 300 Pasteur Drive, Stanford, CA 94305 USA; 2 Department of Cardiovascular Surgery, Osaka University Graduate School of Medicine, 2-2 Yamada-oka, Suita, 565-0871 Japan

**Keywords:** Diabetic cardiomyopathy, Tissue engineering, Cell therapy

## Abstract

**Background:**

Diabetes mellitus is a risk factor for coronary artery disease and diabetic cardiomyopathy, and adversely impacts outcomes following coronary artery bypass grafting. Current treatments focus on macro-revascularization and neglect the microvascular disease typical of diabetes mellitus-induced cardiomyopathy (DMCM). We hypothesized that engineered smooth muscle cell (SMC)-endothelial progenitor cell (EPC) bi-level cell sheets could improve ventricular dysfunction in DMCM.

**Methods:**

Primary mesenchymal stem cells (MSCs) and EPCs were isolated from the bone marrow of Wistar rats, and MSCs were differentiated into SMCs by culturing on a fibronectin-coated dish. SMCs topped with EPCs were detached from a temperature-responsive culture dish to create an SMC-EPC bi-level cell sheet. A DMCM model was induced by intraperitoneal streptozotocin injection. Four weeks after induction, rats were randomized into 3 groups: control (no DMCM induction), untreated DMCM, and treated DMCM (cell sheet transplant covering the anterior surface of the left ventricle).

**Results:**

SMC-EPC cell sheet therapy preserved cardiac function and halted adverse ventricular remodeling, as demonstrated by echocardiography and cardiac magnetic resonance imaging at 8 weeks after DMCM induction. Myocardial contrast echocardiography demonstrated that myocardial perfusion and microvascular function were preserved in the treatment group compared with untreated animals. Histological analysis demonstrated decreased interstitial fibrosis and increased microvascular density in the SMC-EPC cell sheet-treated group.

**Conclusions:**

Treatment of DMCM with tissue-engineered SMC-EPC bi-level cell sheets prevented cardiac dysfunction and microvascular disease associated with DMCM. This multi-lineage cellular therapy is a novel, translatable approach to improve microvascular disease and prevent heart failure in diabetic patients.

## Background

The global prevalence of diabetes mellitus (DM) is rapidly increasing, and patients suffering from this disease have a significantly increased risk of cardiovascular disease and death [1]. According to the American Heart Association, the incidence of coronary artery disease (CAD), myocardial infarction (MI), and death due to MI is higher in diabetic patients than in non-diabetic patients, with at least 68% of diabetic patients over the age of 65 succumbing to some form of heart disease [2]. Furthermore, diabetic patients account for approximately 25% of patients undergoing multi-vessel coronary revascularization in the USA [3]. Unfortunately, DM is a significant risk factor for mortality and repeat coronary revascularization after coronary artery bypass grafting (CABG) or percutaneous coronary intervention (PCI) [3–5]. The adverse impact of DM is even more pronounced in the setting of cardiomyopathy, as patients with low left ventricular ejection fraction (LVEF) do not benefit from CABG when they have coexistent DM [6]. As such, the treatment of CAD in the diabetic heart is challenging.

It is known that DM itself can cause heart failure secondary to diabetic cardiomyopathy (DMCM), which is characterized by combined systolic and diastolic left ventricular dysfunction, increased interstitial fibrosis, and cardiomyocyte apoptosis [7–10]. The pathological mechanisms of DMCM are not fully established, although multiple potential mechanisms have been proposed, including endothelial cell dysfunction, microangiopathy, damage from reactive oxygen species, oxidative stress, and mitochondrial dysfunction [7–10]. Microangiopathy with endothelial cell dysfunction is especially problematic, leading to disruption of coronary vascular autoregulation and subsequent impairment of cardiomyocyte maintenance and survival [11]. Effective therapies to restore the microcirculation are therefore essential, especially in patients with diabetes. Unfortunately, such treatments are lacking, as CABG and PCI may address macrovascular disease but do not ameliorate microvascular disease [11–13].

Cytokine and stem cell-based therapies aimed at micro-revascularization and myocardial repair have been the subject of extensive research, demonstrating encouraging experimental promise. However, subsequent investigation in prospective randomized clinical trials yielded results that were statistically significant but clinically insignificant [14–17]. Limitations of these therapies include individual cytokine/cell-type constraints, transient therapeutic effects secondary to inadequate persistence of treatment agents, lack of a detailed mechanistic understanding, and suboptimal delivery platforms, among others. We have addressed the limitations of cell dispersion, destruction, and isolation in the hostile post-infarction environment with the creation of a tissue-engineered SMC-EPC bi-level cell sheet. The SMC-EPC bi-level cell sheet provides EPCs with a natural SMC biologic support environment, and significantly improves cardiac function in a rodent model of ischemic cardiomyopathy [18–20]. Furthermore, SMC-EPC bi-level cell sheets secrete an abundance of angiogenic cytokines, which directly contribute to the maintenance of a functional microvasculature [18, 21, 22]. Thus, we hypothesized that these tissue-engineered angiogenic constructs may provide an ideal cell and cytokine source for the treatment of diabetic coronary microangiopathy.

In this study, we investigated the therapeutic potential of a novel multi-lineage stem cell construct for the treatment of diabetic coronary microangiopathy. Specifically, we examined to what degree microvascular restoration and myocardial functional recovery occurs, and whether this therapy has translational applications.

## Methods

### Animal care and biosafety

Wistar rats were obtained from Charles River Laboratories, and enhanced green fluorescent protein (EGFP)-transgenic Sprague–Dawley rats were purchased from Rat Resource and Research Center (RRRC). All animals were handled in accordance with the Guide for the Care and Use of Laboratory Animals published by the US National Institutes of Health (NIH Publication No. 85-23, Revised 1996). The experimental protocol was approved by the Institutional Animal Care and Use Committee at Stanford University (Protocol 28921).

### Antibodies

The following primary antibodies were used: α-smooth muscle actin (αSMA), SM22α, CD31, CD34, von Willebrand factor (vWF), GFP, cardiac troponin T, anti-vascular endothelial growth factor receptor-2 (VEGFR-2), transforming growth factor β1 (TGF-β1), TGF-β receptor 1 (TGF-β R1), TGF-β receptor 2 (TGF-β R2), caspase-3, caspase-9, vinculin, mouse IgG polyclonal isotype control, rabbit IgG polyclonal isotype control, and goat IgG polyclonal isotype control (Abcam, Cambridge, MA). Fluorescent-conjugated secondary antibodies included: AlexaFluor^®^488 donkey anti-mouse IgG, AlexaFluor^®^594 donkey anti-rabbit IgG, AlexaFluor^®^594 donkey anti-mouse IgG, AlexaFluor^®^594 donkey anti-goat IgG, AlexaFluor^®^488 donkey anti-rabbit IgG, AlexaFluor^®^488 donkey anti-goat IgG, AlexaFluor^®^594 donkey anti-rabbit IgG, and AlexaFluor^®^647 donkey anti-mouse IgG (Abcam); horseradish peroxidase-conjugated secondary antibody (N-Histofine^®^, NICHIREI Biosciences Inc., Tokyo, Japan); 4′ 6-diamidino-2-phenylindole (DAPI, NucBlue^®^ Fixed Cell ReadyProbes^®^ Reagent, ThermoFisher Scientific, Waltham, MA).

### Isolation of MSCs and EPCs, and MSC differentiation to SMCs

**Fig. 1:**
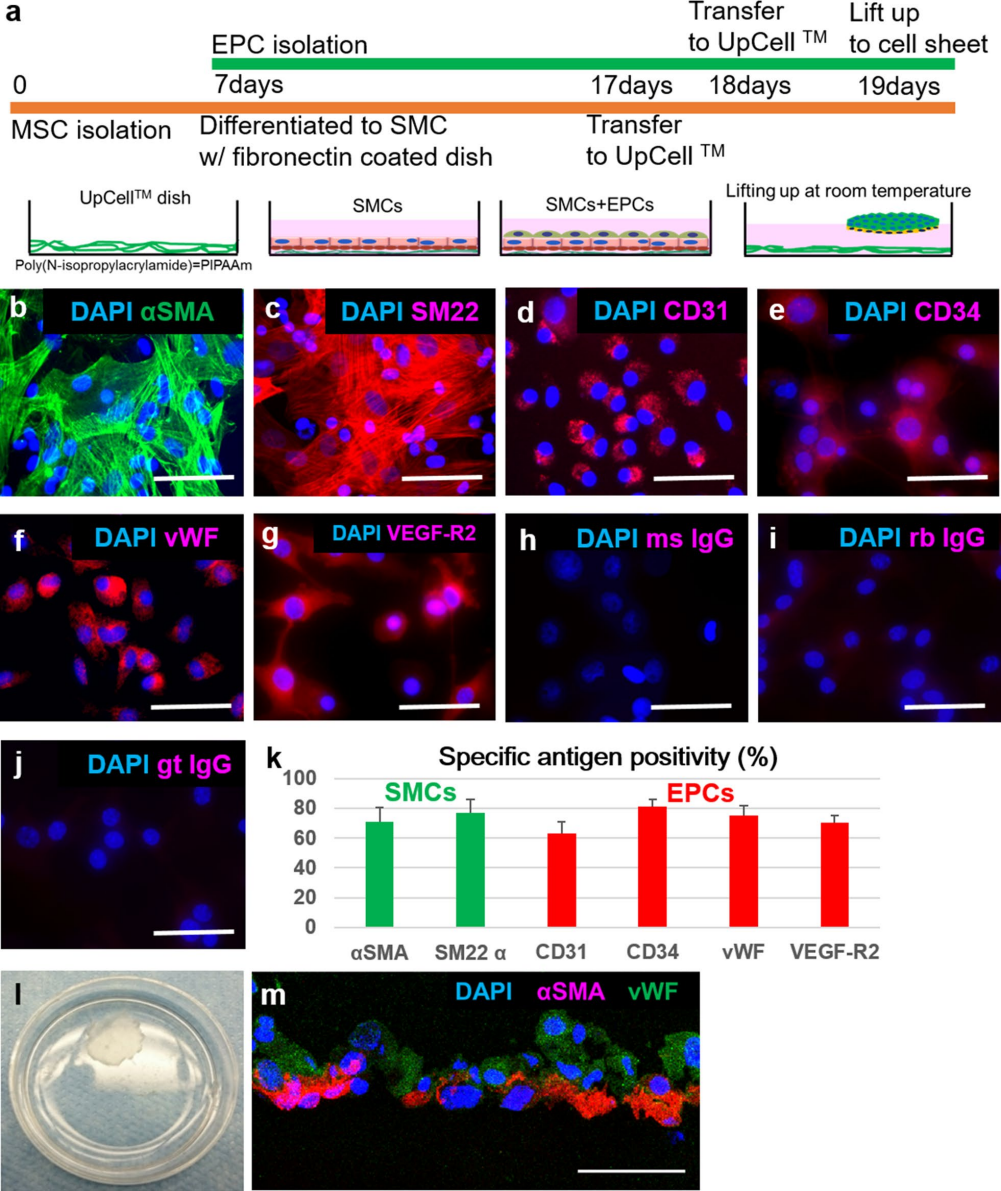
Characterization of SMCs, EPCs, and EPC-SMC bi-level cell sheets. **a** SMC-EPC bi-level cell sheet manufacturing protocol. **b**–**g** Immunocytochemistry demonstrated αSMA and SM22α on SMCs (**b**, **c**), and CD31, CD34, vWF, and VEGF-R2 on EPCs (**d**–**g**). Images of isotype controls of mouse, rabbit, and goat IgG were provided (**h**–**j**). Scale bar = 50 µm. **k** Percentages of each antigen for SMCs and EPCs were high, and demonstrated that our protocol yielded SMCs and EPCs with high purity. **l** A round-shaped scaffold-free SMC-EPC bi-level cell sheet in a 35 mm-dish. **m** Immunostaining of the SMC-EPC bi-level cell sheet with anti-vWF (green) and anti-αSMA (red) antibodies. The cell nuclei were counterstained with DAPI (blue). Scale bar = 50 µm. *EPC* endothelial progenitor cell, *SMC* smooth muscle cell, *MSC* mesenchymal stem cell, *DAPI* 4′,6-diamidino-2-phenylindole, *αSMA* α smooth muscle actin, *sm22α* smooth muscle protein 22-α, *vWF* von Willebrand factor, *VEGF-R2* vascular endothelial growth factor-receptor 2, *ms IgG* mouse immunoglobulin G, *rb IgG* rabbit immunoglobulin G, *gt IgG* gout immunoglobulin G

Rat MSCs were obtained from male Wistar rats (8 weeks old, 250–300 g; Charles River, San Jose, CA), as previously described [18, 19]. Briefly, bone marrow (BM) mononuclear cells were isolated from the long bones, filtered through a 40 μm cell strainer, and centrifuged at 300*g* for 8 min. Red blood cells (RBCs) were excluded using 1x RBC lysis buffer (eBioscience, Inc., San Diego, CA) for 10 min at 4 °C. The remaining cells were cultured in DMEM (Gibco, Thermo Fisher Scientific) with 10% FBS on non-coated culture dishes at 37 °C and 5% CO_2_. The adherent cells were cultured for 1 week. Then, the primary MSCs were transferred to fibronectin-coated dishes (Corning^®^ Biocoat™, Discovery Labware, Inc., Bedford, MA) at a density of 5 × 10^3^ cells/cm^2^ to induce differentiation into SMCs (Fig. 1a). The SMC-differentiation protocol yielded approximately 5 × 10^6^ cells from 2 rat donors.

EPCs were isolated and cultured as described previously [18–20]. Briefly, BM mononuclear cells were isolated from the long bones of Wistar rats and cultured on vitronectin-coated dishes (Sigma-Aldrich, St. Louis, MO) in EBM-2 supplemented with EGM-2 SingleQuot (Lonza, Walkersville, MD). EGFP-labeled EPCs were isolated from transgenic Sprague–Dawley rats (SD-Tg(UBC-EGFP)2BalRrrc) using the same procedure to investigate EPC fate tracking.

To characterize isolated and differentiated cells, immunocytochemistry for αSMA, SM22α, CD31, CD34, vWF, and VEGFR-2 was performed. We seeded the cells in a chamber slide (Lab-Tek™ II Chamber Slide™ System; 4-well, Nunc, Rochester, NY), and 3 wells were used for each antibody. Five fields were randomly selected and 5 images per well were acquired at a magnification of 200x. Positivity of the SMC- or EPC-specific markers in the cultured cells was determined from the acquired images using computer-based cell counting with Image J (National Institutes of Health, Bethesda, MD). Cells were counterstained with DAPI, and assessed for nuclei with fluorescent microscopy (Leica DMi8, Leica Microsystems Inc., Buffalo Grove, IL). The percentage of cells with SMC- or EPC-specific markers was calculated in each image by the following formula: (number of specific marker-positive cells/number of nuclei) × 100%. The average percentage of the 5 images indicated the degree of positivity of SMC- or EPC-specific markers per well, and these experiments and analyses were performed in triplicate.

### Creation of SMC-EPC bi-level cell sheets

When SMCs reached 80–90% confluence, they were detached using a trypsin–EDTA solution and plated at a density of 1.0 × 10^6^ cells/dish on 35 mm temperature-responsive culture dishes (UpCell™, Cellseed, Tokyo, Japan). After being cultured at 37 °C with 5% CO_2_ for 24 h, EPCs were detached with a trypsin–EDTA solution and collected using a cell scraper before being re-plated at a density of 1.0 × 10^6^ cells/dish on UpCell™ culture dishes (Cellseed) that were already confluent with SMCs. The dishes were incubated at room temperature, which caused the cells to detach spontaneously and form SMC-EPC bi-level cell sheets (Fig. 1a).

For characterization, a SMC-EPC bi-level cell sheet was immunolabeled with anti-αSMA and anti-vWF antibodies. Confocal microscopy (SP8 inverted confocal, Leica Microsystems Inc.) with a DAPI counterstain was used for analysis.

### Induction of rat DMCM model and cell sheet transplant

Female Wistar rats were randomized into 3 groups: control (no DM induction with sham surgery, n = 11), untreated DMCM (DM induction with sham surgery, n = 13), and treated DMCM (DM induction treated with SMC-EPC cell sheet implantation through a left thoracotomy, n = 13). Under anesthesia, a single intraperitoneal injection of streptozotocin (STZ; 60 mg/kg, Sigma-Aldrich) was administered to the DMCM groups to induce diabetes, whereas the control group received the same volume of PBS injected intraperitoneally. One week following STZ administration, blood glucose levels were measured to confirm hyperglycemia (> 300 mg/dl). Rats in the DMCM groups with blood glucose levels < 300 mg/dl were excluded.

**Fig. 2:**
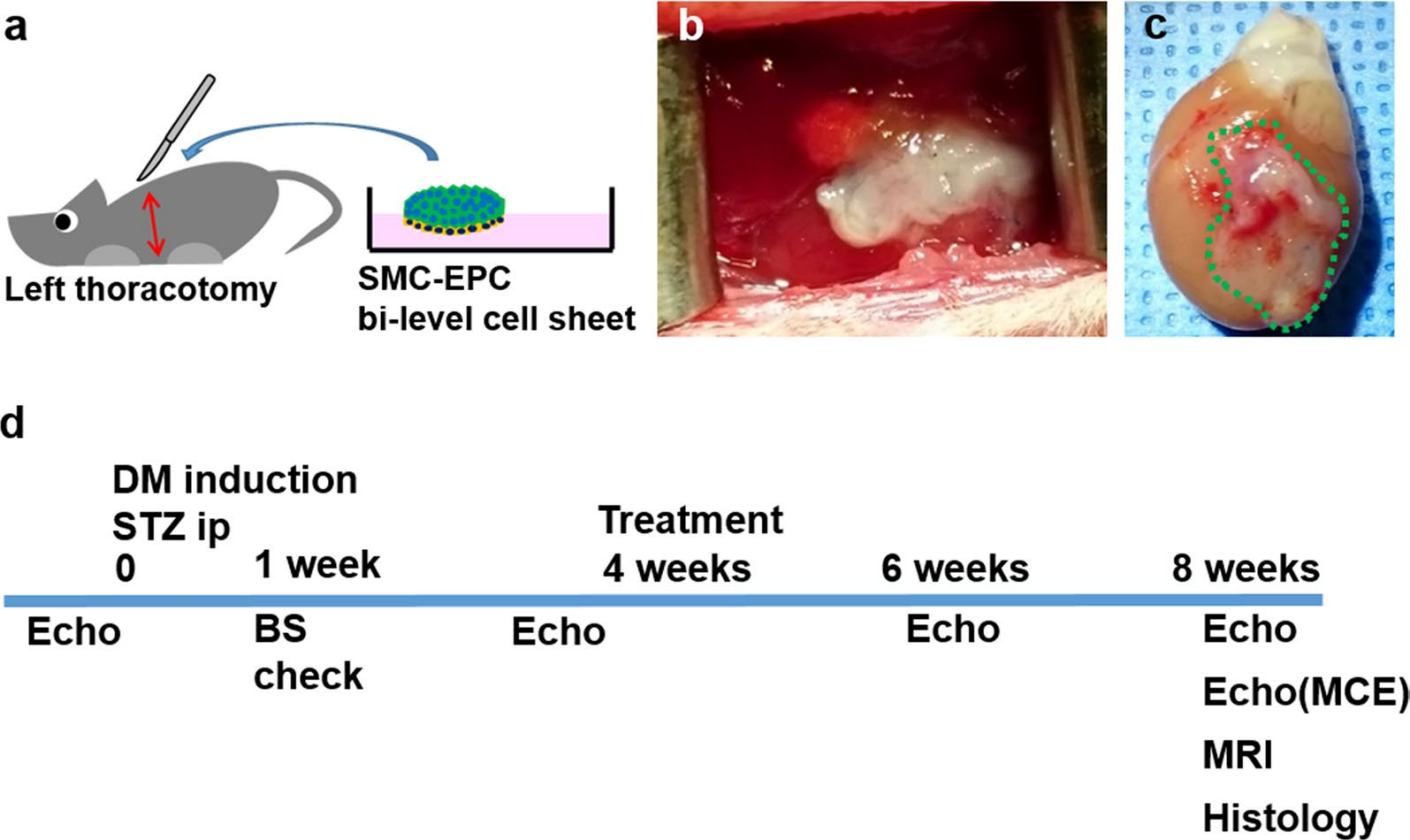
Experimental technique and protocol timeline. **a** Schema illustrating SMC-EPC bi-level cell sheet implantation technique. **b** The cell sheet broadly covered the left ventricular anterior wall from the base to apex. **c** The cell sheet remained on the heart, visualized directly via explant 1 week after transplant. **d** Timeline of experimental protocol. *SMC* smooth muscle cell, *EPC* endothelial progenitor cell, *DM* diabetes mellitus, *STZ* streptozotocin, *echo* echocardiography, *BS* blood sugar, *MCE* myocardial contrast echocardiography, *MRI* magnetic resonance imaging

Surgery was performed 4 weeks following STZ or PBS injection. A left thoracotomy was performed and animals in the treated DMCM group underwent SMC-EPC bi-level cell sheet implantation prior to chest closure whereas animals in the control group and untreated DMCM groups underwent chest closure with no further procedures (Fig. 2a). The SMC-EPC bi-level cell sheet broadly covered the entire anterior wall of the left ventricle (LV) (Fig. 2b). We put small stitches with 8-0 polypropylene suture on the epicardium of the anterior wall, taking care to avoid any coronary arteries. The cell sheet was overlaid onto the stitches and hooked with the knots of the suture to prevent the cell sheet from slipping away. In the fate tracking experiments described below, we confirmed that the cell sheet remained fixed on the heart at 1 week after transplant (Fig. 2c). Eight weeks after DM induction (4 weeks after surgery), following the completion of cardiac MRI and echocardiography, the animals were humanely killed and the heart was explanted for analysis (Fig. 2d).

To investigate EPC and SMC fate tracking, we created the SMC-EPC bi-level cell sheet using EGFP-labeled EPCs and SMCs from male rat donors. After the induction of diabetes in 5 Sprague–Dawley female rats, the cell sheet transplant was performed using the same technique as described above. Five weeks after DM induction (1 week following implantation), the animals were humanely killed for histological analysis.

### Echocardiography

Echocardiography was performed at time 0 (immediately prior to DM induction), at 4 weeks after DM induction but prior to surgery, at 6 weeks after DM induction (2 weeks after surgery), and at 8 weeks after DM induction (4 weeks after surgery) for each group; control n = 11, untreated n = 13, treated n = 13 (Fig. 2d). All images were acquired on a Vevo Imaging Station (VisualSonics Inc., Toronto, Canada) equipped with a Vevo 2100 system (VisualSonics Inc.) and ultra-high frequency liner array transducer (MicroScan™ MS250 13–24 MHz transducer, VisualSonics Inc.). All analyses and measurements were performed with Vevo LAB software (VisualSonics Inc.) including left ventricular end-diastolic diameter (LVEDD), left ventricular end-systolic diameter (LVESD), and fractional shortening (FS) [23].

### LV volumetry with cardiac MRI

Eight weeks after DM induction (4 weeks following surgery), cardiac MRI was performed for each group using a 7 Tesla small animal MRI system; control n = 11, untreated n = 13, treated n = 13 (BioSpec USR70/30 7T, Bruker Corporation, Fremont, CA). Data acquisition was performed with a 4-channel phased array receive-only surface coil (Rapid MR International, Columbus, OH) placed around the chest. Respiratory and ECG monitoring were performed using an MRI-compatible physiological monitoring system (SA Instruments, Inc., Stony Brook, NY). Cine short axis cardiac images were acquired using a Bruker FcFLASH sequence. Two- and 4-chamber long axis views of the heart were obtained to define the short axis image orientation. All data was stored in DICOM format and images were transferred to OsiriX Lite (OsiriX, Bernex, Switzerland) for analysis of left ventricle end-systolic volume (LVESV, µl), left ventricle end-diastolic volume (LVEDV, µl), and LVEF (%).

### Myocardial contrast echocardiography

Myocardial contrast echocardiography (MCE) was performed at 8 weeks after DM induction (4 weeks after surgery) to evaluate relative myocardial perfusion for each group; control n = 8, untreated n = 11, treated n = 12. A 24-gauge intravenous catheter was inserted into a tail vein and microbubble contrast agent (Untargeted Vevo MicroMaker, microbubble concentration; 1 × 10^7^/50 µl, 200 µl injection per rat, VisualSonics Inc.) was injected. The same imaging system and transducer as described above was used for image acquisition. Data was acquired on the end-systolic phase with ECG and respiratory double-gating during the procedure. Nonlinear contrast agent imaging mode was used for quantification of relative myocardial perfusion. First, the study was performed with the animal at rest. After 20 min, a vasodilator stress study was performed using a selective adenosine A2A agonist (CGS-21680, 5 µg/kg, Abcam), and data acquisition and contrast agent injection was performed 5 min following vasodilator administration. VevoCQ software (VisualSonics Inc.) was used for data analysis following the user manual. The region of interest (ROI) in each image was manually positioned within the myocardium and in the adjacent LV cavity. The peak enhancement in the myocardium, indicating relative blood volume, was normalized by referencing the peak enhancement in the nearby LV cavity [24]. Peak enhancement at rest and in the vasodilated stress state were compared.

### Histology

Following heart explant after the study end-point, each rat heart specimen (control n = 10, untreated n = 11, treated n = 11) was divided into 3 parts: the base, middle, and apex. The apex part was used for protein extraction and Simple Western analysis as described below. The base part was fixed with 10% buffered formalin and embedded in paraffin for permanent section, and the middle part was frozen in liquid nitrogen for frozen section analysis. The paraffin-embedded sections were stained with picrosirius red to assess interstitial fibrosis. Ten fields of the anterior LV myocardium were randomly selected in each section at a magnification of 200x, and relative fibrosis was measured as the percentage of red-stained area using ImageJ. The frozen sections were immunolabeled with anti-CD31 antibody. Ten fields of the anterior LV myocardium at 400x magnification from each section were randomly selected, and the number of stained vascular endothelial cells per field were manually counted using a light microscope. We distinguished capillaries from arterioles based on the size, defining a cut-off lumen diameter of 15 μm, and we only counted vessels < 15 μm size with positive immunohistochemistry. The number of stained blood vessels from the 10 fields were averaged, and the results were expressed as vascular density per mm^2^. We also used frozen sections for EPC and SMC fate tracking after EGFP-labeled cell sheet transplant. EPCs were detected with EGFP signal, and frozen sections were immunolabeled with anti-GFP, anti-vWF, and anti-αSMA antibodies. SMCs were detected using fluorescence in situ hybridization (FISH) to the sex-determining region Y (sry) gene. We used a red-labeled FISH probe specific to the sry gene (Rat IDetectTM chromosome paint probe; Empire Genomics, Buffalo, NY), and followed the user manual. After FISH, the sections were immunolabeled with anti-αSMA antibody. Confocal microscopy (SP8 inverted confocal, Leica Microsystems Inc.) with a DAPI counterstain (ThermoFisher Scientific) was used for analysis.

### Measurement of survival rate among transplanted cells with quantitative PCR

To assess the survival rate of the EPC-SMC bi-level cell sheet after transplant, we measured the copy number of sex-determining region Y (sry) gene and brain-derived neurotrophic factor (bdnf) gene with a fluorescent-based quantitative PCR using the ViiA™ 7 Real-Time PCR system (Applied Biosystems, Foster city, CA), as described previously [25]. Briefly, we isolated both EPCs and SMCs from male rat donors, and created the EPC-SMC bi-level cell sheet with a total 2 × 10^6^ cells (1 million cells each). Sequentially, these were transplanted onto the female diabetic heart. Whole hearts were explanted to determine the survival rates at 5 min, 1 week, and 4 weeks after transplant (n = 3 each). In addition, to describe a standard curve, EPCs and SMCs from male rat donors were mixed in 1:1 ratio, and injected onto explanted female diabetic whole hearts with known numbers: 0, 2, 2 × 10, 2 × 10^2^, 2 × 10^3^, 2 × 10^4^, 2 × 10^5^, or 2 × 10^6^ cells (n = 3 each). Samples were homogenized, and genomic DNA was prepared with Easy-DNA™ kit (invitrogen, Carlsbad, CA). TaqMan^®^ Gene Expression Assays for sry (Rn04224592_u1, Applied Biosystems) and bdnf (Rn02531967_s1, Applied Biosystems) genes were performed following the user instructions, and a total amount of 1.0 μg DNA was applied for each assay. All measurements were performed in triplicate. The standard plot was used to determine the number of surviving cells from the percentage of male cells. Specifically, the estimated survival rate of the transplanted cells was calculated by the following formula: 2 × (sry copy number/bdnf copy number) × 100%.

### Protein extraction and Simple Western analysis

Proteins were extracted from the apex part of explanted hearts (control n = 10, untreated n = 11, treated n = 11) using a radio-immunoprecipitation assay (RIPA, Sigma-Aldrich) buffer with a protease and phosphatase inhibitor (Thermo Scientific™). Using 50 µl of RIPA buffer with protease and phosphatase inhibitor, samples were lysed and the supernatant was separated. Protein concentrations in the lysates were measured with a Bradford assay (Biorad), and the concentration of each sample was optimized for Simple Western assay (0.4–1.0 mg/ml) for each antibody.

Simple Western analyses (Wes Simple Western Analysis, ProteinSimple) were performed per the user manual. The Simple Western assay is a capillary electrophoresis technique that automates protein loading, separation, immunoprobing, washing, and detection, allowing for absolute protein quantitation [26]. Sample lysates were quantified as the peak area by the Compass software per the ProteinSimple protocols. All experiments in Simple Western analysis were performed in triplicate. Primary antibodies against VEGF, TGF-β1, TGF-β receptor 1, TGF-β receptor 2, caspase-3, caspase-9, and vinculin were used.

### Statistical analyses

Statistical analysis was performed using IBM SPSS Statistics 24 (IBM Corporation, Armonk, NY). Data are expressed as mean ± standard deviation. The Kruskal–Wallis test was used for comparisons between 3 groups, followed by the post hoc pairwise Wilcoxon rank-sum test for comparisons between groups or the Wilcoxon signed-rank test for comparisons within groups. Cardiac function by echocardiography was assessed by repeated-measures analysis of variance with group, time, and group × time interaction effects. All probability values were 2-sided, and values of P < 0.05 were considered statistically significant.

## Results

### Characterization of EPCs, and SMC differentiation from MSCs

The protocol used to manufacture SMC-EPC bi-level cell sheets is illustrated in Fig. 1a. EPC- or SMC-specific markers were used to characterize the cell cultures and verify cell sheet composition, which was consistent with previous papers [19, 27–30]. After MSC-to-SMC differentiation, the cells were highly positive for αSMA (70.8 ± 9.8%) and SM22α (76.9 ± 9.1%, Fig. 1b, c, k). EPCs cultured for 11 days after isolation were positive for CD31 (62.9 ± 8.3%), CD34 (81.1 ± 4.8%), vWF (75.0 ± 6.9%), and VEGF receptor-2 (70.2 ± 4.7%) on their surface (Fig. 2d–g, k). Our protocol yielded SMCs and EPCs with high purity from bone marrow, which is a promising cell source for translatable clinical applications. Next, we created circular, scaffold-free SMC-EPC bi-level cell sheets in temperature-responsive culture dishes, which allowed for spontaneous detachment of the cell sheet from the culture surface after 10–20 min of incubation at room temperature (Fig. 1l). We confirmed that the cell sheet was bi-level by immunohistochemistry (Fig. 1m).

### Recovery of cardiac function following SMC-EPC bi-level cell sheet transplant in DMCM rat model

**Fig. 3:**
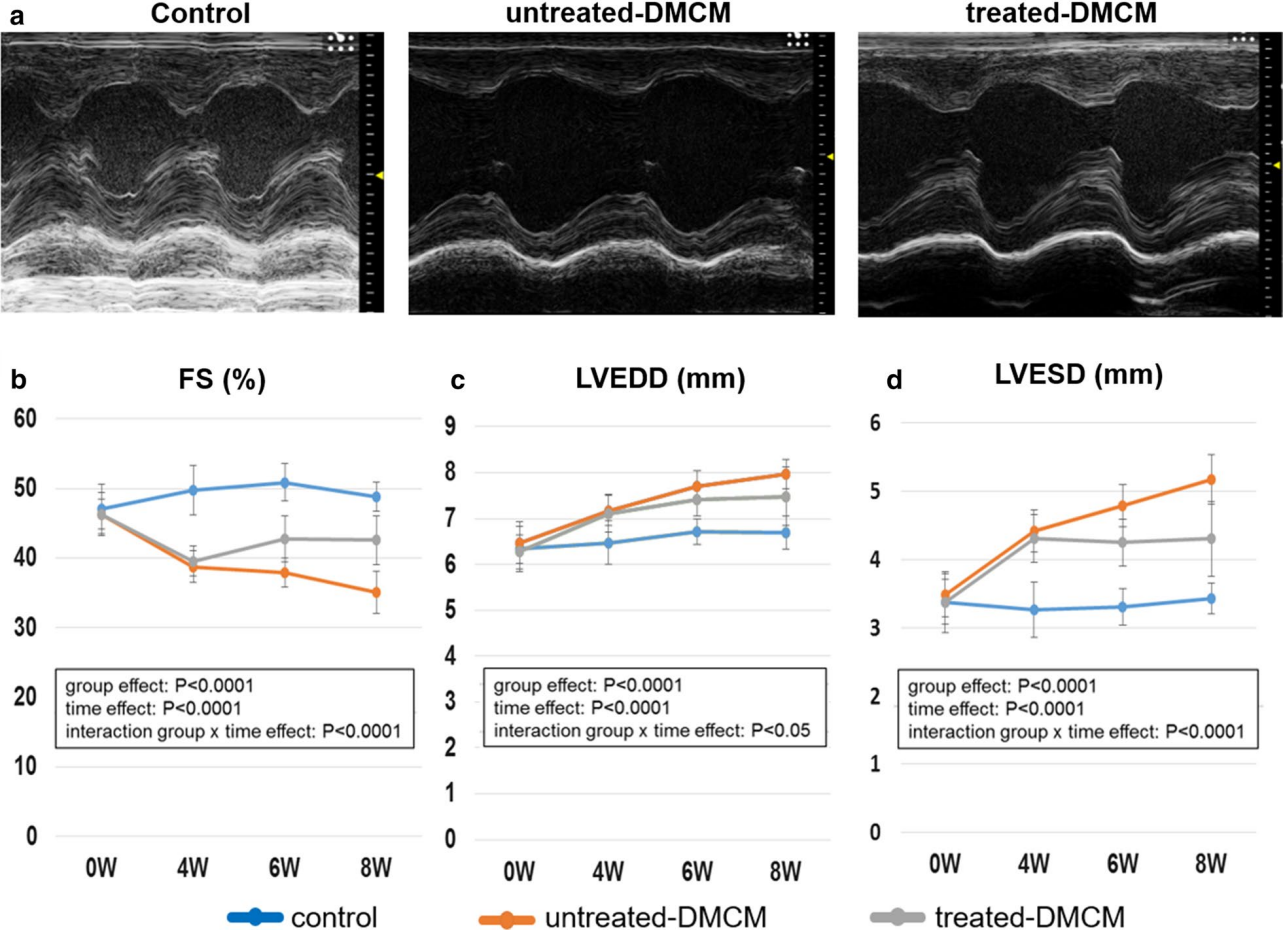
Echocardiographic analysis demonstrates functional improvements following cell sheet implantation. **a** Representative M-mode images at 4 weeks post-surgery. **b**–**d** In the animals treated with cell sheet implantation, left ventricular fractional shortening (**b**) was significantly higher and both left ventricular end-diastolic diameter (**c**) and left ventricular end-systolic diameter (**d**) were significantly reduced as compared to the untreated group. *FS* fractional shortening, *LVEDD* left ventricular end-diastolic diameter, *LVESD* left ventricular end-systolic diameter

To evaluate cardiac function after treatment, we performed serial standard transthoracic echocardiography (Fig. 1b). Pre-treatment parameters did not differ significantly between the untreated and treated groups. Untreated DMCM rats demonstrated an upward trend in LVEDD and LVESD, and a downward trend in LVFS prior to treatment (Fig. 3b–d). At both 6 and 8 weeks following DM induction (2 and 4 weeks after surgery), LVFS was significantly greater in the treated DMCM group than in the untreated DMCM group (6 weeks 42.7 ± 3.3% vs. 37.9 ± 2.0%; 8 weeks 42.6 ± 3.5% vs. 35.1 ± 3.0%, P < 0.0001, Fig. 3b and Table 1). The LVEDD and LVESD were significantly smaller in the treated DMCM group than in the untreated DMCM group both at 6 and 8 weeks following DM induction (2 and 4 weeks after treatment), as shown in Table 1 (P < 0.05 for LVEDD and P < 0.0001 for LVESD for interaction effect of time and group in the repeated ANOVA, Fig. 3c, d and Table 1).

**Table 1:** Echocardiographic data

	0 (Pre-DM induction)	4 weeks (Pre-treatment)	6 weeks (2 weeks after treatment)	8 weeks (4 weeks after treatment)
FS (%)				
Control	47.0 ± 3.6	49.8 ± 3.5	50.9 ± 2.7	48.8 ± 2.1
Untreated DMCM	46.3 ± 2.2	38.8 ± 2.3	37.9 ± 2.7	35.1 ± 3.0
Treated DMCM	46.3 ± 3.1	39.5 ± 2.2	42.7 ± 3.3	42.6 ± 3.5
Group effect: P < 0.0001; time effect: P < 0.0001; interaction group × time effect: P < 0.0001				
LVEDD (mm)				
Control	6.338 ± 0.497	6.477 ± 0.481	6.711 ± 0.276	6.694 ± 0.359
Untreated DMCM	6.471 ± 0.456	7.173 ± 0.327	7.705 ± 0.341	7.965 ± 0.320
Treated DMCM	6.273 ± 0.379	7.114 ± 0.414	7.405 ± 0.352	7.483 ± 0.635
Group effect: P < 0.0001; time effect: P < 0.0001; interaction group × time effect: P < 0.05				
LVEDD (mm)				
Control	3.368 ± 0.449	3.262 ± 0.449	3.305 ± 0.268	3.427 ± 0.268
Untreated DMCM	3.477 ± 0.315	4.416 ± 0.306	4.786 ± 0.309	4.786 ± 0.362
Treated DMCM	3.373 ± 0.330	4.305 ± 0.348	4.242 ± 0.347	4.300 ± 0.550
Group effect: P < 0.0001; time effect: P < 0.0001; interaction group × time effect: P < 0.0001				

Serial data for fraction shortening (%), left ventricle end-diastolic dimension (mm), and left ventricle end-systolic dimension (mm) were demonstrated

*DM* diabetes mellitus, *DMCM* diabetic cardiomyopathy, *FS* fraction shortening, *LVEDD* left ventricle end-systolic dimension, *LVESD* left ventricle end-systolic dimension

**Fig. 4:**
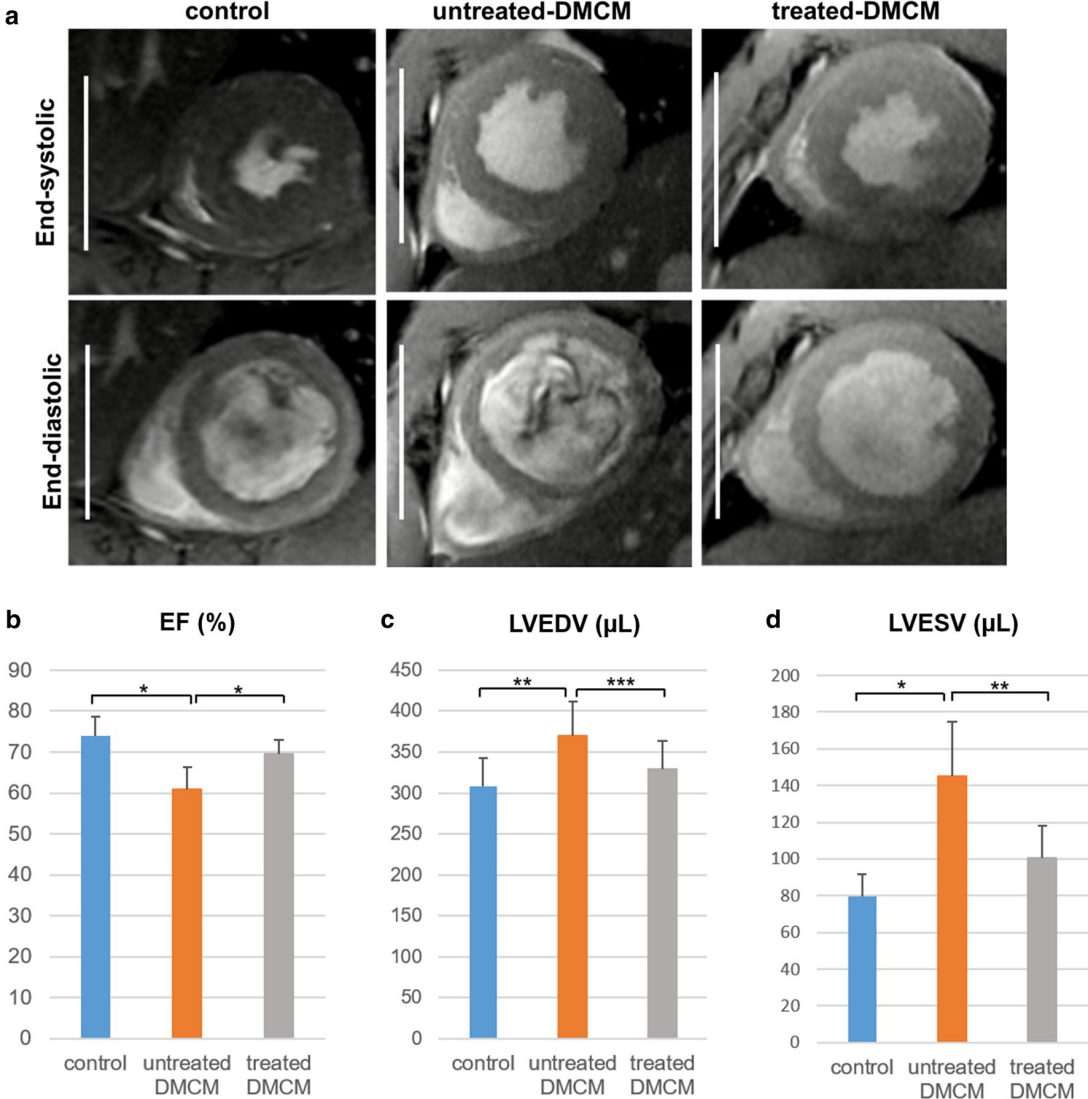
Cardiac magnetic resonance imaging reveals preserved left ventricular geometry following cell sheet therapy. **a** Representative cardiac MRI images at end-systolic and end-diastolic phase for each experimental group. **b**–**d** In the cell sheet treatment group, left ventricular ejection fraction (**b**) was significantly improved and both left ventricular end-diastolic volume (**c**) and left ventricular end-systolic volume (**d**) were significantly reduced as compared to the untreated group. Scale bars, 10 mm; *P < 0.001, **P < 0.01, ***P < 0.05. *EF* ejection fraction, *LVEDV* left ventricular end-diastolic volume, *LVESV* left ventricular end-systolic volume

We also performed cardiac MRI to determine LV volumetry at 8 weeks after DM induction (4 weeks following treatment), with representative cardiac MRI images at end-systolic and end-diastolic phases shown in Fig. 4a. The treatment group demonstrated significantly higher LVEF (69.4 ± 3.6% vs. 60.9 ± 5.4%, P < 0.001, Fig. 4b), with significantly reduced LVEDV (329 ± 35 µl vs. 370 ± 41 µl, P < 0.05, Fig. 4c) and LVESV (101 ± 17 µl vs. 145 ± 30 µl, P < 0.01, Fig. 4d).

### Myocardial contrast echocardiography demonstrated improved microvascular function after SMC-EPC bi-level cell sheet implantation

**Fig. 5:**
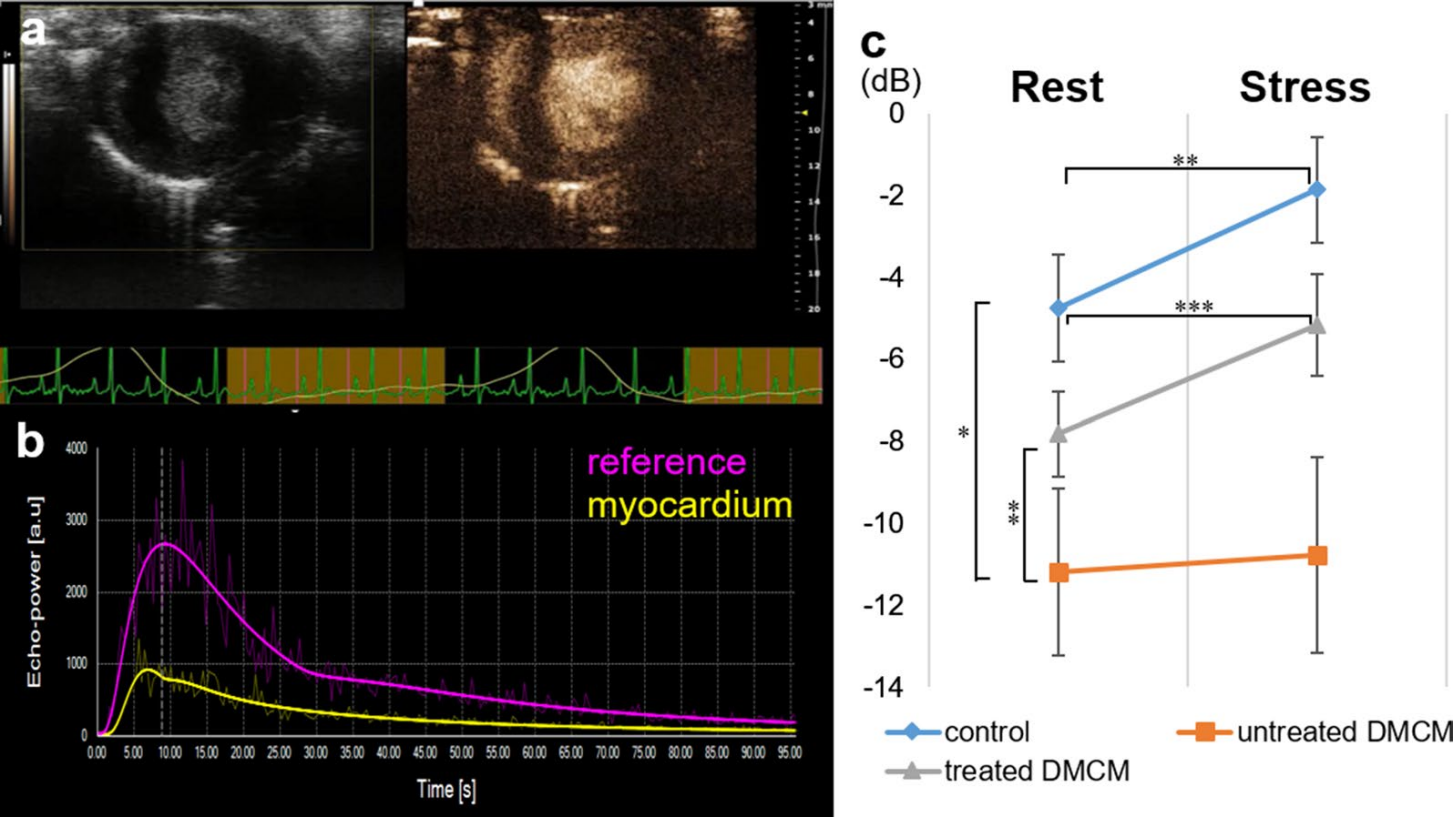
Myocardial contrast echocardiography shows improved microvascular function after cell sheet implantation. **a** images at 4-week end-point with a B-mode image on the left and a nonlinear contrast image on the right. **b** Typical video intensity graph generated during imaging. **c** At rest, peak video intensity in untreated diabetic animals was significantly lower than in nondiabetic controls and animals treated with cell sheet implantation. Following vasodilator-induced stress, peak video intensity was unchanged in the untreated group, whereas the nondiabetic control and cell sheet-treated groups demonstrated increased peak video intensities. *P < 0.001, **P < 0.05, ***P < 0.01

DM results in functional deterioration of the coronary circulation and adverse coronary arteriolar structural remodeling [31]. MCE is a useful tool to estimate myocardial blood flow (MBF) at rest and during stress in small animals [32, 33]. Myocardial flow reserve, which is the increase in MBF during stress compared with MBF at rest, is a reliable measure of coronary endothelial function [34]. We evaluated myocardial perfusion and microvascular function using MCE. A representative image of the procedure and ROI peak video intensity graph are shown in Figs. 5a and b. Signal enhancements were measured both at rest and after vasodilator-induced stress to evaluate microvascular function (Fig. 5c). At rest, peak video intensity in the untreated group was significantly lower than in the nondiabetic group (− 11.2 ± 2.0 dB vs. − 4.8 ± 1.3 dB, P < 0.001, Fig. 5c). A significant improvement in peak video intensity was noted in the treatment group compared with the untreated group (− 7.8 ± 1.0 dB vs. − 11.2 ± 2.0 dB, P < 0.05, Fig. 5c), indicating that cell sheet implantation preserved myocardial perfusion in diabetic cardiomyopathy. After vasodilator-induced stress, peak video intensity was unchanged in the untreated group (− 10.8 ± 2.4 dB vs. − 11.2 ± 2.0 dB, P = 0.657, Fig. 5c), whereas peak video intensities increased with vasodilator-induced stress in both the nondiabetic control (− 1.9 ± 1.3 dB vs. − 4.8 ± 1.3 dB, P < 0.05, Fig. 5c) and cell sheet-treated group (−5.2 ± 1.2 dB vs. − 7.8 ± 1.0 dB, P < 0.01, Fig. 5c), suggesting preserved microvascular function in the cell sheet treatment group.

### SMC-EPC bi-level cell sheet implantation decreased interstitial fibrosis and increased microvascular density

**Fig. 6:**
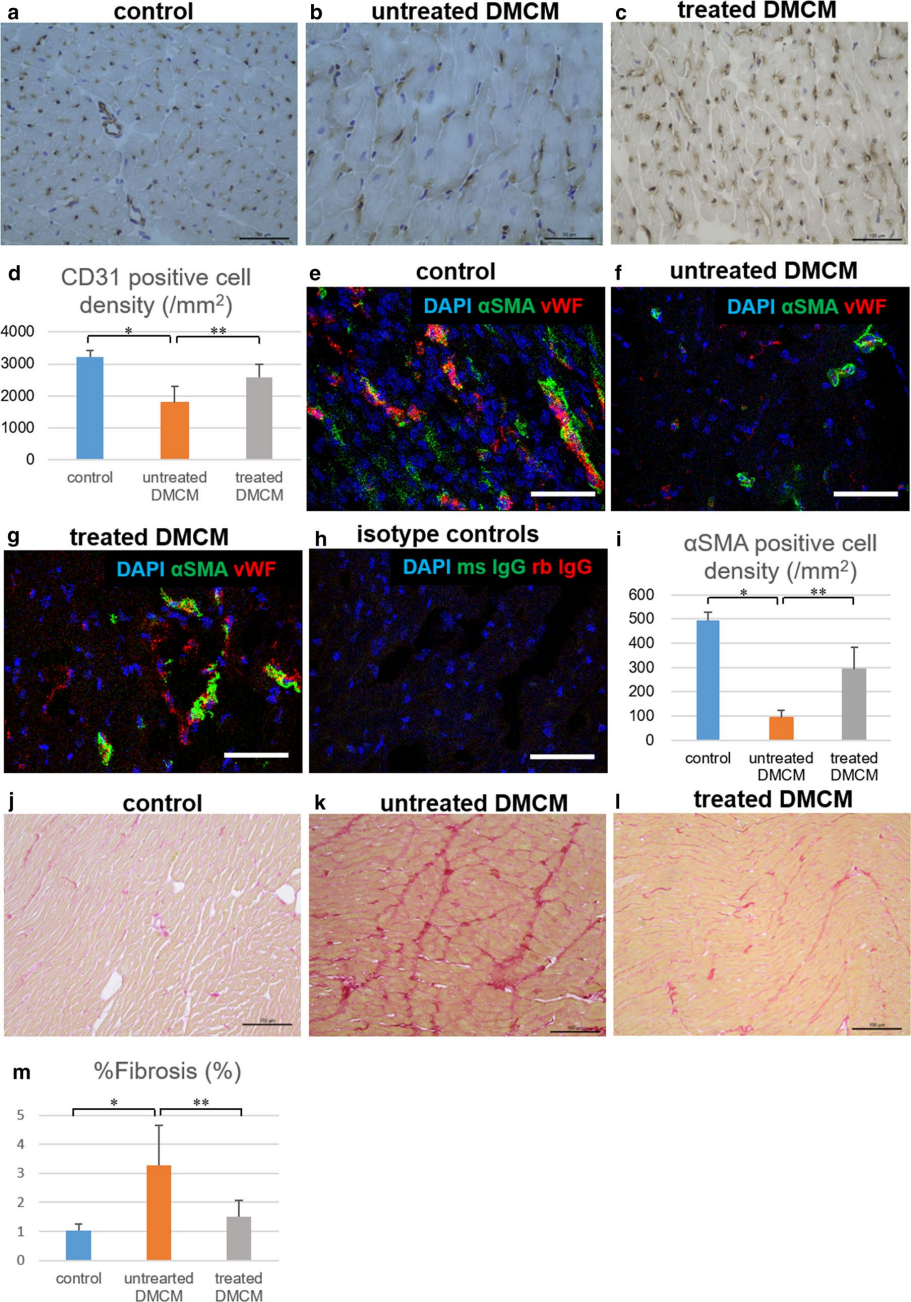
Histological evaluation reveals increased microvascular density and decreased interstitial fibrosis following SMC-EPC bi-level cell sheet implantation with successful EPC integration. **a**–**i** Microvascular density at 4-week study end-point in the control group (**a**, **e**), untreated group (**b**, **f**), and cell sheet-treated group (**c**, **g**) assessed semi-quantitatively by immunohistochemistry for CD31 and αSMA showed significantly enhanced microvascular density in the cell sheet-treated group (**d**, **i**). Image of isotype controls was also provided (**h**). **j**–**m** Semi-quantitative evaluation of interstitial fibrosis in the control group (**j**), untreated group (**k**), and cell sheet-treated group (**l**) demonstrates significantly less interstitial fibrosis in animals treated with cell sheet implantation as compared to untreated animals (**m**). Image scale bars, 100 µm in **a**–**c**, **j**–**l**, 50 µm in **e**–**h**; *P < 0.001, **P < 0.05. *αSMA* alpha smooth muscle actin, *CD31* cluster of differentiation-31, *DAPI* 4′,6-diamidino-2-phenylindole, *DMCM* diabetic cardiomyopathy, *ms IgG* mouse immunoglobulin G, *rb IgG* rabbit immunoglobulin G, *vWF* von Willebrand factor

Increased interstitial fibrosis, cardiomyocyte apoptosis, and microangiopathy are potential underlying pathologic mechanisms for DMCM [7–10]. We examined explanted heart specimens 8 weeks after DM induction (4 weeks following surgery) to characterize microvascular density with immunohistochemistry for CD31 (Fig. 6a–d) and αSMA (Fig. 6e–i), and pathological interstitial fibrosis by picrosirius red staining (Fig. 6j–m). The density of CD31-positive cells in the cell sheet treatment group was significantly greater than that in the untreated group (2573 ± 412 vessels/mm^2^ vs. 1777 ± 522 vessels/mm^2^, P < 0.05, Fig. 6d), and the density of αSMA-positive cells in the treated group was significantly higher than that in the untreated group (93 ± 29 vessels/mm^2^ vs. 293 ± 89 vessels/mm^2^, P < 0.05, Fig. 6i). The cell sheet treatment group also exhibited significantly less interstitial fibrosis than the untreated group (1.5 ± 0.5% vs. 3.3 ± 1.4%, P < 0.05, Fig. 6m).

### Fate tracking and the survival rate of transplanted EPCs and SMCs

**Fig. 7:**
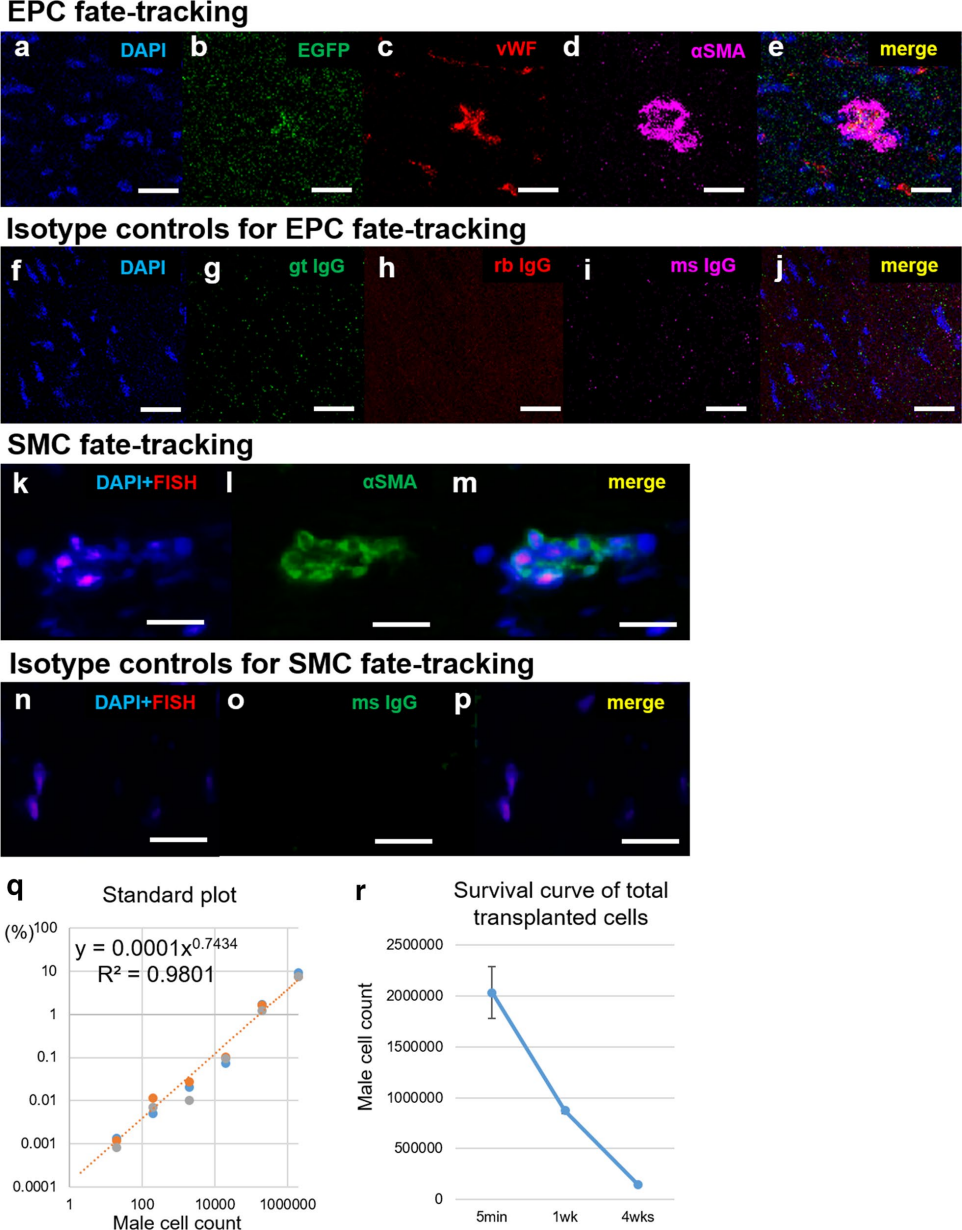
Fate tracking and survival rate of transplanted cells. **a**–**j** Fate tracking was performed with EGFP-labeled EPCs. EGFP and vWF double-positive cells were detected in the specimens 1 week after implantation (**a**–**e**). Images of isotype controls were provided (**f**–**j**). **k**–**p** SMCs were tracked with FISH for sry gene of male cells. Y-chromosome and αSMA double-positive cells were also detected in the specimens 1 week after implantation (**k**–**m**), and images of isotype controls were provided (**n**–**p**). **q**–**r** To assess the survival rate of the EPC-SMC bi-level cell sheet after transplant, the copy number of sry gene of male cells was measured with a fluorescent-based quantitative PCR. Samples with known numbers of male transplanted cells produced a standard plot with high correlation coefficient (r^2^ = 0.980, **q**). A survival curve of total transplanted cells was then generated (**r**). Image scale bars, 10 µm in **a**–**j**, 20 µm in **k**–**p**. *αSMA* alpha smooth muscle actin, *EGFP* enhanced green fluorescent protein, *DAPI* 4′,6-diamidino-2-phenylindole, *FISH* fluorescence in situ hybridization, *gt IgG* goat immunoglobulin G, *ms IgG* mouse immunoglobulin G, *rb IgG*, rabbit immunoglobulin G, *vWF* von Willebrand factor

SMC-EPC bi-level cell sheets secrete angiogenic factors and have potent paracrine effects [20]. In addition, these cell sheets have the ability to integrate into host vasculature and contribute to repair in an ischemic cardiomyopathy model [20]. We performed a fate tracking experiment to confirm that the same phenomenon occurs in DMCM. We used EGFP-labeled EPC bi-level cell sheets, with EPCs isolated from the bone marrow of EGFP transgenic rats. One week after the transplant, EGFP and vWF double-positive cells were detected in the host myocardium, suggesting that the transplanted EPCs migrated and integrated into the host vasculature in DMCM (Fig. 7a–e). In addition, sry gene-positive cells were found in female rat hearts by FISH, and these cells were also αSMA positive (Fig. 7k–m). This indicates that transplanted SMCs also directly contribute to the formation of new vessels in the host myocardium.

We also calculated the survival rate of cells within the EPC-SMC bi-level cell sheet after transplant using quantitative PCR for the sry gene. The correlation coefficient of the standard curve was 0.980, determined by using quantitative PCR for heart samples with a known number of male cells injected (Fig. 7q). After transplant of cell sheets containing 2 × 10^6^ cells, the number of surviving cells were calculated using the standard curve to be 59,177 ± 56,657 cells (33 ± 4.9%) at 1 week, and 6652 ± 6334 cells (3.7 ± 0.2%) at 4 weeks (Fig. 7r).

### SMC-EPC bi-level cell sheet implantation results in increased angiogenic factors, decreased fibrosis, and reduced apoptosis

**Fig. 8:**
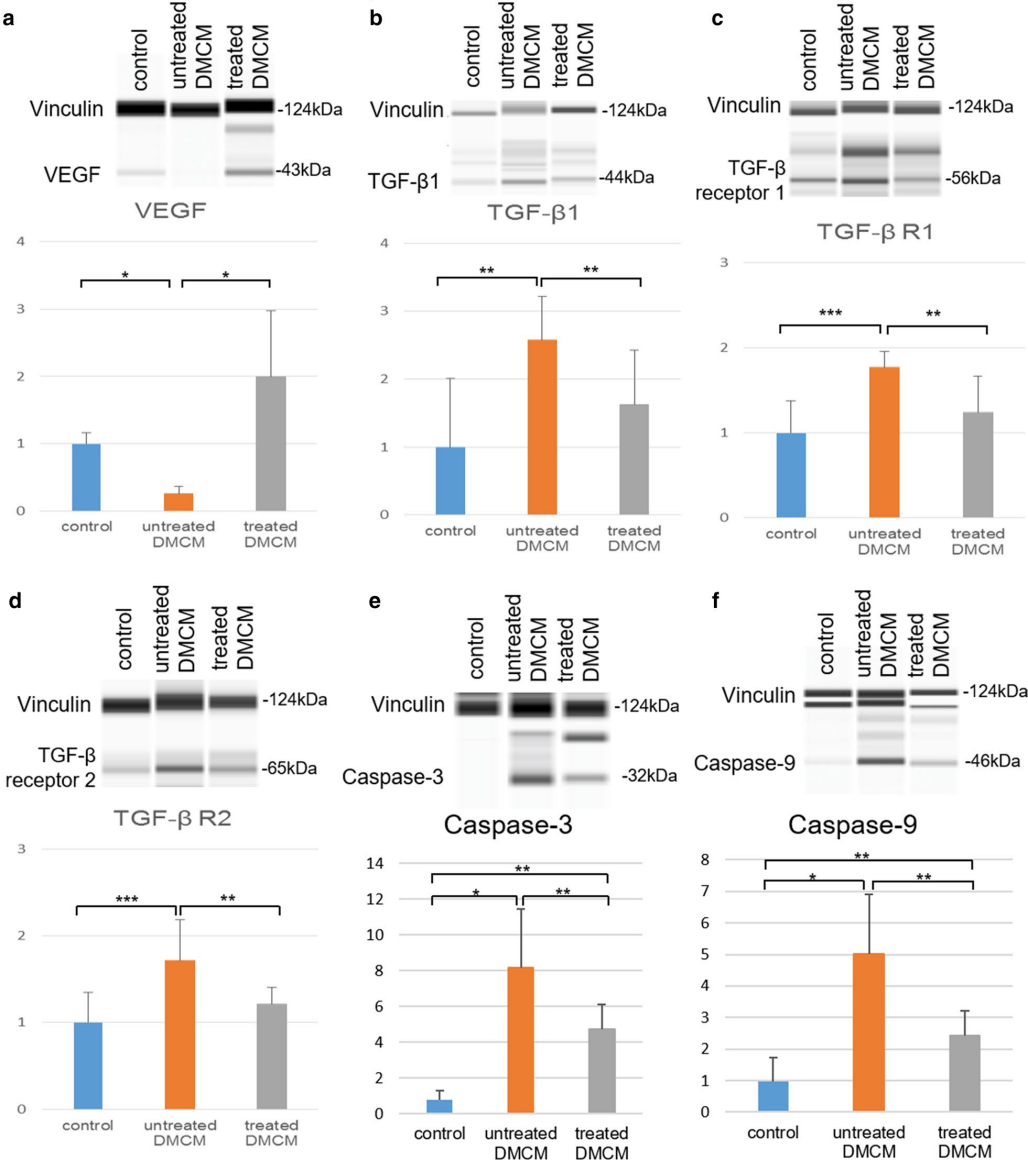
Simple Western assay demonstrates increased levels of pro-angiogenic cytokines and decreased markers of fibrosis and apoptosis 4 weeks following EPC-SMC bi-level cell sheet implantation. **a** VEGF protein expression was decreased in the untreated diabetic group with significantly higher relative VEGF expression in the cell sheet treatment group as compared to untreated animals. **b**–**d** TGF-β1, TGF-β receptor 1, and TGF-β receptor 2 protein expression was increased in the untreated diabetic group with significantly higher relative expression of TGF-β1, TGF-β receptor 1, and TGF-β receptor 2 in the untreated animals than in the cell sheet treatment group. **e**–**f** Caspase-3 and caspase-9 protein expression were increased in the untreated animals with significantly lower relative expression in the cell sheet treated group as compared to the untreated group. *P < 0.001, **P < 0.05, ***P < 0.01

Prior research demonstrated that the pathological changes observed in diabetic cardiomyopathy are associated with VEGF downregulation [35]. To examine the underlying mechanisms of the cell sheet therapy, we characterized VEGF protein expression in tissue samples 8 weeks after DM induction (4 weeks after surgery) using the Simple Western assay. VEGF protein expression was decreased in the untreated group, and the relative expression level of VEGF was significantly higher in the treatment group as compared with the untreated group (2.00 ± 0.98 vs. 0.26 ± 0.98, P < 0.001, data presented relative to control, Fig. 8a), suggesting enhanced angiogenesis in the cell sheet treatment group. Furthermore, increased interstitial fibrosis is implicated in the development of diabetic cardiomyopathy [7–10]. We demonstrated that the expression of TGF-β1 and TGF-β receptor 1 and 2 were increased in the untreated group, with significantly higher relative expression of TGF-β1, TGF-β receptor 1, and TGF-β receptor 2 in the untreated group as compared with the treatment group (TGF-β1: 2.57 ± 0.64 vs. 1.62 ± 0.81, P < 0.05; TGF-β R1: 1.77 ± 0.18 vs. 1.24 ± 0.42, P < 0.05; TGF-β R2: 1.71 ± 0.46 vs. 1.21 ± 0.19, P < 0.05, data presented relative to control, Fig. 8b–d), suggesting decreased fibrosis in the treated DMCM group. Finally, coronary microangiopathy with endothelial cell dysfunction is associated with DMCM, and eventually leads to myocardial ischemia and cardiomyocyte apoptosis [7–10]. We studied the effect of EPC-SMC bi-level cell sheet implantation on cardiomyocyte apoptosis using caspase-3 and caspase-9 protein expression as a surrogate for apoptosis. Caspase-3 and caspase-9 protein relative expression were increased in the untreated group and lower in the treatment group (caspase-3: 4.72 ± 1.40 vs. 8.15 ± 3.28, P < 0.05, data presented relative to control, Fig. 8e; caspase-9: 2.42 ± 0.81 vs. 5.02 ± 1.88, P < 0.05, data presented relative to control, Fig. 8f), suggesting decreased apoptosis in the group treated with EPC-SMC bi-level cell sheet implantation.

## Discussion

The results of this study suggest that SMC-EPC bi-level cell sheet implantation can attenuate the deleterious functional effects of DMCM in a rodent model. In addition, SMC-EPC bi-level cell sheet therapy also appears to preserve microvascular function through pro-angiogenic, anti-fibrotic, and anti-apoptotic properties, as suggested by increased VEGF, decreased TGF-β1 and TGF-β receptor, and decreased caspase-3 and caspase-9 expression, respectively. Histological analysis supports this supposition, as demonstrated by increased microvascular density, diminished interstitial fibrosis, and integration of EPCs and SMCs into host vasculature.

EPCs exist in both the bone marrow and circulating peripheral blood, playing a key role in the regulation of endothelial cell function and vascular homeostasis [27, 36, 37]. When endothelial cell injury occurs secondary to hypoxia or trauma, EPCs are released from the bone marrow, travel to the site of injury, and repair the vasculature through cytokine-mediated tissue activation, integration, and angiogenesis [27]. Circulating EPCs also participate in this repair pathway. In the setting of diabetes, however, the number of circulating EPCs is reduced and bone marrow microangiopathy causes EPC apoptosis in a rodent model, which may contribute to the development of diabetic microvascular disease and DMCM [38, 39]. In a study of human patients suffering from diabetes, oxidative stress and advanced glycation end-products caused decreased EPC function due to cell injury [40, 41]. These studies suggest that in diabetes, the stem cell niche of bone marrow might be structurally and functionally impaired, resulting in decreased bone marrow EPC release and diminished peripheral EPC mobilization, which ultimately hinders the repair of endothelial cell injury.

Our therapy addresses these issues directly by restoring the local EPC population through SMC-EPC bi-level cell sheet implantation, which prevented the development of cardiovascular dysfunction in our rodent model of DMCM. The cell sheet transplant is a local therapy, whereas DMCM affects the heart globally. Despite this issue, we demonstrated the effectiveness of SMC-EPC bi-level cell sheet therapy in DMCM, and we believe that our method delivered enough cells to treat broad regions of myocardium, leading to improvement of global cardiac function. Interestingly, VEGF expression was preserved in animals treated with cell sheet implantation as compared to untreated animals, which may explain the higher microvascular density found in the treatment group. In addition, we previously demonstrated that transplanted EPCs and SMCs integrated into the host vasculature and exerted therapeutic effects both directly and via a paracrine-mediated pathway in a rat model of acute MI [18, 20]. There were fewer newly-forming vessels containing transplanted cells in our rat DMCM model than in our MI model, and the cell survival rate at 4 weeks was approximately 3.7% in the DMCM model. Nevertheless, our treatment still resulted in decreased myocardial apoptosis and interstitial fibrosis in DMCM, although there are large differences in the underlying disease mechanisms between MI and DMCM. Overall, our strategy of expanding EPCs ex vivo and transferring large numbers of these cells to the heart via a cell sheet appears to be a reasonable method of replenishing local EPCs, and preventing the development of DMCM and diabetic microvascular dysfunction through the above mechanisms.

Recent studies have shown that forkhead box transcription factor 1 (FOXO1) plays an important role in the pathogenesis of DMCM [42]. The FOXO1 pathways involve inflammation, oxidative stress, nitrosative stress, glucose metabolism, lipid metabolism, autophagy, apoptosis, and hypertrophy-related gene expressions, but also alter cardiac structure/function and metabolism and promote cardiac cell death during the development and progression of DMCM [42]. Whether FOXO1 gene expression can be influenced after SMC-EPC bi-level cell sheet transplant in a DMCM model represents an interesting topic of future study.

It has been reported that systemically-injected bone marrow-derived EPC therapy improves cardiac function, inhibits cardiomyocyte apoptosis, and attenuates myocardial fibrosis in a rodent DMCM model [28]. This suggests that EPCs themselves may have potent healing potential, though adjunctive strategies to promote the efficacy of EPC therapy have also been described. Preconditioning EPCs with diazoxide, a highly selective mitochondrial ATP-sensitive K+ channel opener, enhances EPC survival under oxidative and hyperglycemic stress, which potentiates their treatment effect in DMCM [30]. In a hindlimb ischemia model, co-administration of EPCs and smooth muscle progenitor cells (SMPCs) increased therapeutic efficacy through the formation of a stable vascular network [43]. The interaction between endothelial cells and SMCs appears to be critical in the maturation and stabilization of vessels, and while paracrine signaling plays a role, direct contact between the two cell types is essential [44, 45]. As double-layer constructs, our tissue-engineered SMC-EPC bi-level cell sheets preserve this critical cell–cell interaction both by paracrine signaling and direct contact. By maintaining physiological conditions and cell–cell interactions, our strategy appears to facilitate vessel formation and stabilization, and may be a useful method to enhance the efficacy of EPC therapy.

Our MSC differentiation protocol gave rise to SMCs with approximately 70% purity. The remainder of the cells could include residual MSCs. MSCs are known to produce and secrete cytokines and growth factors that contribute to paracrine activities. These factors demonstrate effects on cardiomyocyte regeneration and angiogenesis, and also have anti-fibrosis, anti-apoptotic activity, and anti-inflammatory properties [46]. It has been reported that MSC injection inhibits myocardial apoptosis in DMCM [47]. In addition, MSCs demonstrated greater anti-inflammatory effects and restored diastolic function more effectively than EPCs for hypertensive cardiomyopathy, using a porcine renovascular hypertension model [48]. This indicates that MSCs have different mechanisms to treat a failing heart. Therefore, we acknowledge the possibility that residual MSCs could have cardioprotective effects in our diabetic model, and may have partially contributed to the preservation of cardiac function in the present study.

## Conclusion

In conclusion, implantation of tissue-engineered SMC-EPC bi-level cell sheets attenuated cardiac dysfunction and preserved microvascular function in DMCM. This multi-lineage angiogenic therapy is a novel and potentially translatable approach to treat microvascular disease and prevent heart failure in patients with diabetes.

## Abbreviations

αSMA: alpha smooth muscle actin
bdnf: brain-derived neurotrophic factor
BM: bone marrow
CABG: coronary artery bypass grafting
CAD: coronary artery disease
CD31: cluster of differentiation-31
CD34: cluster of differentiation-34
DAPI: 4,6-diamidino-2-phenylindole
DICOM: Digital Imaging and COmmunications in Medicine
DM: diabetes mellitus
DMCM: diabetic cardiomyopathy
DMEM: Dulbecco’s Modified Eagle Medium
EBM-2: endothelial basal medium-2
ECG: electrocardiogram
EDTA: ethylenediaminetetraacetic acid
EGFP: enhanced green fluorescent protein
EGM-2: endothelial growth medium-2
EPC: endothelial progenitor cell
FBS: fetal bovine serum
FISH: fluorescence in situ hybridization
FOXO1: forkhead box transcription factor 1
FS: fractional shortening
gt IgG: goat immunoglobulin G
IgG: immunoglobulin G
LV: left ventricle
LVEDD: left ventricle end-diastolic diameter
LVEDV: left ventricle end-diastolic volume
LVEF: left ventricular ejection fraction
LVESD: left ventricle end-systolic diameter
LVESV: left ventricle end-systolic volume
MBF: myocardial blood flow
MCE: myocardial contrast echocardiography
MI: myocardial infarction
MRI: magnetic resonance imaging
MSC: mesenchymal stem cell
ms IgG: mouse IgG
PBS: phosphate buffered saline
PCR: polymerase chain reaction
rb IgG: rabbit IgG
RBC: red blood cell
PCI: percutaneous coronary intervention
RIPA: radio-immunoprecipitation assay
ROI: region of interest
RRRC: Rat Resource and Research Center
SMC: smooth muscle cell
SMPC: smooth muscle progenitor cell
sm22α: smooth muscle protein 22-alpha
sry: sex-determining region Y
STZ: streptozotocin
TGF-β: transforming growth factor–beta1
TGF-β R1: transforming growth factor-beta receptor 1
TGF-β R2: transforming growth factor-beta receptor 2
VEGFR-2: vascular endothelial growth factor receptor-2
vWF: von Willebrand factor
